# Utility of histopathological revision in the management of gastro-entero-pancreatic neuroendocrine neoplasia

**DOI:** 10.1007/s12020-023-03418-3

**Published:** 2023-06-20

**Authors:** Matteo Marasco, Ludovica Magi, Evelina Rogges, Elisabetta Dell’Unto, Maria Rinzivillo, Emanuela Pilozzi, Bruno Annibale, Francesco Panzuto

**Affiliations:** 1 Digestive Disease Unit, ENETS Center of Excellence, Sant’Andrea University Hospital, Sapienza University of Rome, Rome, Italy; 2 Department of Translational and Precision Medicine, Sapienza University of Rome, Rome, Italy; 3 Pathologic Morphological and Molecular Anatomy Unit, Sant’Andrea University Hospital, Department of Clinical and Molecular Medicine, Sapienza University of Rome, Rome, Italy; 4 Department of Medical-Surgical Sciences and Translational Medicine, Sapienza University of Rome, Rome, Italy

**Keywords:** Neuroendocrine tumors, Ki67, Histology, Multidisciplinary team

## Abstract

**Background:**

Histological evaluation and grading assessment are key points in the diagnostic work-up of gastroentero-pancreatic neuroendocrine neoplasms (GEP-NENs).

**Aim:**

To analyze the impact of histopathological revision on the clinical management of patients with GEP-NEN.

**Materials and methods:**

Patients referred to our Center of Excellence between 2015 and 2021 were included in this study. Immunohistochemical slides at the time of initial diagnosis were reviewed to assess tumor morphology, diagnostic immunohistochemistry, and Ki67.

**Results:**

101 patients were evaluated, with 65 (64.4%) gastrointestinal, 25 (24.7%) pancreatic, and 11 (10.9%) occult neoplastic lesions suspected to be of GEP origin. The main changes resulting from the revision were: first Ki-67 assessment in 15.8% of patients, Ki-67 change in 59.2% of patients and grading modification in 23.5% of patients. An additional immunohistochemical evaluation was performed in 78 (77.2%) patients, leading to a confirmation of GEP origin in 10 of 11 (90.9%) of unknown primary site neoplastic lesions and an exclusion of NEN diagnosis in 2 (2%) patients. After histopathological revision, a significant modification in clinical management was proposed in 42 (41.6%) patients.

**Conclusions:**

Histopathological revision in a referral NEN center is strongly advised in newly diagnosed GEP-NENs to properly plan prognostic stratification and therapeutic choice.

## Introduction

Gastro-entero-pancreatic (GEP) neuroendocrine neoplasms (NENs) are rare and heterogeneous diseases arising from the diffuse neuroendocrine system of the digestive system, including tumors of the gastrointestinal tract (GI NEN) and the pancreas (pNEN) [1]. Owing to the improved use of radiology and endoscopic procedures, their incidence is increasing over time, accounting for ~0.5% of new malignancy diagnoses [2, 3]. Their prognosis is affected by several factors, including primary tumor site, staging, and grading expressed by Ki-67 index assessment [4]; the latter is considered the strongest predictor for clinical outcomes, providing useful information for tailored treatment. Thus, histopathological evaluation of tissue specimens, including grading assessment, is a crucial point in the diagnostic work-up of these neoplasms. The European Neuroendocrine Tumor Society (ENETS) guidelines specify that a pathological report of GEP-NENs should include morphology and differentiation on hematoxylin/eosin (HE) section, immunostaining for neuroendocrine markers by synaptophysin (Syn) and chromogranin A (CgA) and, once the neuroendocrine nature of the tumor is established, the proliferative activity must be assessed preferably by using Ki-67 staining [5]. Based on the proliferative activity and tumor differentiation, NEN may be classified into well-differentiated (NET G1: Ki67 < 3%, NET G2: Ki67: 3–20%, and NET G3: Ki67 > 20%) and poorly differentiated NEC G3 [6]. This last group is further subclassified into small cell and large cell types. Indeed, a Ki67 level of >55% has been further proposed to identify those diseases with a higher response to chemotherapy and worse prognosis compared to those with a lower proliferative activity (<55%) [7, 8].

Up to 40% of NENs are metastatic at diagnosis, with lesions predominantly found in the liver; the primary tumor site cannot be found by routine imaging or histopathology in approximately 10% of cases, leading to challenging management and a worse prognosis [9]. In the setting of a diagnosis of NEN with an unknown primary site, immunostaining of other diagnostic markers may be used, including hormones (insulin, gastrin, serotonin) and transcription factors (TTF-1, CDX2, Isl-1) [5]: in fact, CDX2 positivity is expressed in the primary small bowel intestine, and Isl-1 is expressed primarily in the pancreas and duodenum [5, 10].

To date, surgery is considered the only curative option in NEN, even in cases of locoregional disease; however, selected patients with limited disease or favorable histopathological criteria may be managed by a noninvasive approach by endoscopic follow-up or clinical observation, depending on the primary tumor site [11]. Although histopathological evaluation is well known to be the cornerstone in the prognostic evaluation and management of GEP-NEN [12–14], a proportion of patients referred to dedicated-NEN centers still require histopathological revision [15].

In this study, we aimed to analyze the impact of histopathological revision performed in an ENETS Center of Excellence on clinical management in GEP-NEN patients.

## Materials and methods

This is a retrospective analysis of consecutive patients referred between January 2015 and December 2021 to the Sant’Andrea University Hospital after the center was certified as Center of Excellence by the ENETS in 2014. In accordance with the centers’ standard procedures and following the ENETS guidelines, all major clinical and pathological data were collected in an anonymized database.

All patients were discussed in a NEN multidisciplinary team (MDT) meeting, and a pathological revision was required in those patients for whom the available histological information was not in accordance with the ENETS standards of care [5, 15].

The exclusion criteria were primary tumor site other than GEP or unknown (with exception of those suspected to be GEP based on radiological imaging or pathological information) and lack of the first histopathological report.

We decided to include patients in whom the primary tumor site was not known if the tumors were believed to derive from the small intestine due to the presence of a carcinoid syndrome or in nonfunctioning tumors as a result of the histological criteria as reported in other studies [16]. The pathological revision was performed on site by a pathologist with extensive experience in NEN disease (EP); the immunohistochemical slides at the time of initial NEN diagnosis were obtained from paraffin-embedded tissue blocks and reviewed to assess tumor morphology, diagnostic immunohistochemistry, Ki67 and tumor grade in accordance with the ENETS Consensus Guidelines [5, 17]. All the main histopathological information (Ki-67, tumor grade, differentiation, immunohistochemical markers, microscopic invasion) was collected both from the initial report and the revision. When needed, additional immunohistochemical markers were assessed to confirm the neuroendocrine phenotype or in cases of unknown primary tumor sites suspected to be of GEP origin. The clinical impact after histological revision was considered when the NEN-dedicated MDT proposed a change in therapeutic management after pathological re-evaluation. The distribution of continuous variables was reported as the median and range, and qualitative variables were reported as frequencies and percentages. Subgroups were compared using Fisher’s exact test or the chi-square test, as appropriate. To evaluate the clinical impact of histologic revision, logistic regression analysis was performed to identify the variables associated with changes in clinical management after histological revision. The *P* value was considered significant when it was <0.05. Statistical analysis was performed by MedCalc^®^ v.17 software (MedCalc Software, www.medcalc.org). Full informed consent for data collecting was obtained from the participating patients. The study was conducted according with the declaration of Helsinki. Given the study design (retrospective/observational) no ethical approval is required.

## Results

A total of 125 patients, for whom after the MDT discussion the available histological information was not in accordance with the ENETS standards of care [5], were evaluated for study inclusion. Of these, 14 patients were excluded because the initial pathological report was lacking, and 10 patients were excluded because the primary tumor site was not of GEP origin. Thus, the final analysis was performed on 101 patients, with 65 (64.4%) gastrointestinal (15 ileal, 15 rectal, 15 stomach, 10 appendix, 5 colon, 4 duodenal, 1 extrapancreatic biliary tract), 25 (24.7%) pancreatic, and 11 (10.9%) occult neoplastic lesions suspected to be of GEP origin. The general features of the population included are summarized in Table 1.

**Table 1:** General features of the population (101 patients) at time of initial diagnosis

Feature	N of pts
Male gender	43 (42.6%)
Median age at diagnosis (range)	59 yr (18–88)
Primary tumor site	
Gastrointestinal	65 (64.4 %)
Pancreas	25 (24.7%)
Unknown primary	11 (10.9%)
Tumor stage	
I	32 (31.7%)
II	13 (12.9%)
III	17 (16.8%)
IV	39 (38.6%)
Pathological specimen	
Surgical or endoscopic resection	48 (47.5%)
Biopsy	53 (52.5%)
Site of pathological specimen	
Primary tumor	69 (68.3%)
Metastases	32 (31.7%)
Liver	26 (25.7%)
Other	6 (5.9%)
Median Ki-67^a^ (range)	4 (1–95)
Grading^b^	
G1	34 (40%)
G2	40 (47%)
G3	11 (13%)
Tumor differentiation^c^	
Well differentiated	60 (80%)
Moderately differentiated	10 (13.3%)
Poorly differentiated	5 (6.7%)
Available immunohistochemical staining	46 (45.5%)
CgA	38 (37.6%)
SYN	32 (31.7%)
CK	8 (7.9%)
CDX2	9 (8.9%)
CD56	10 (9.9%)

^a^Not available in 20 pts

^b^Not available in 16 pts

^c^Not available in 26 pts

At the time of initial diagnosis, 39 (38.6%) patients had stage IV disease with metastases predominantly found in the liver (92.3%); 3 (7.7%) had peritoneal metastases.

**Fig. 1:**
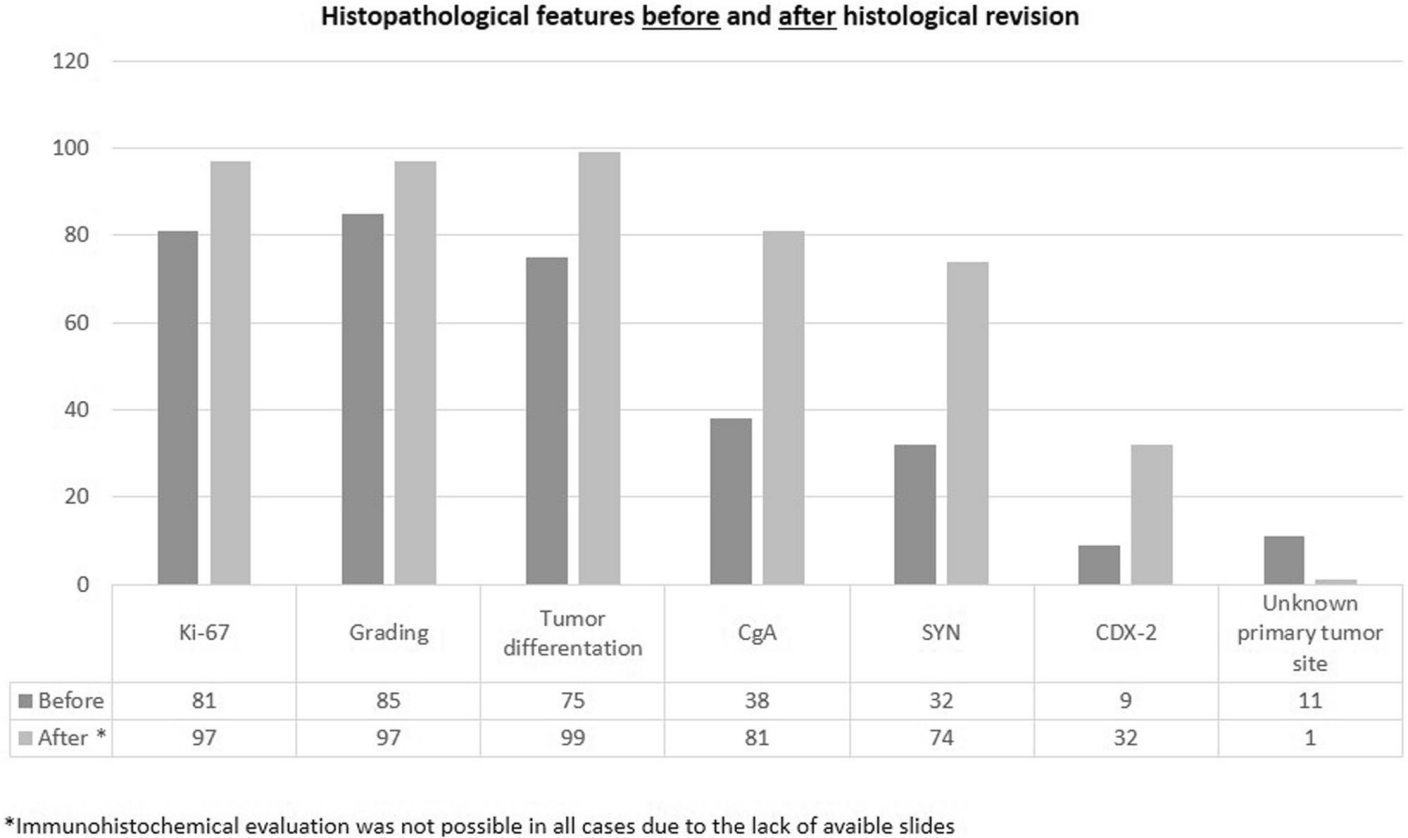
Main pathological tumor features available before and after the hitological revision at the center was performed

When checking the available charts before performing histological revision, the following data were reported in the histological reports referring to the initial diagnosis: tumor differentiation in 75 (74.2%) cases, specific immunohistochemical assessment by CgA and Syn in 38 (37.6%) and 32 (31.7%) cases, respectively, grading in 85 cases (81.2%), and Ki67 evaluation in 81 (80.2%) samples. After histological revision, tumor morphology and grading were available in almost all patients (Fig. 1).

The main histopathological changes resulting from the revision are summarized in Table 2. A modification of the Ki-67 index occurred in 48 patients (59.2%) with an overall decrease in the median value (from 4% to 2%); Ki-67 reassessment changed the grading classification in 20 patients (23.5%) with predominant downstaging from G2 to G1 occurring in 16.5% of cases; and the Ki-67 index was first assessed in 16 (15.8%) patients (in the remaining 4 patients, Ki67 assessment was not feasible due to the lack of available histological slides required to perform grading evaluation).

**Table 2:** Main histopathological findings available after histological revision

Feature	*N* of pts
Ki-67 index^a^	
Change in Ki-67 value	48 (59.2%)
Median Ki-67 after revision	2 (1–95)
Grading change^b^	
Overall	20 (23.5%)
G1→G2	2 (2.3%)
G2→ G3	1 (1.2%)
G3→ G2	3 (3.5%)
G2→G1	14 (16.5%)
Tumor differentiation change^c^	
Overall	11 (14.7%)
Moderately differentiated→well differentiated	10 (13.3%)
Poorly differentiated→well differentiated	1 (1.3%)
Additional immunohistochemical staining	
Overall	78 (77.2%)
CgA	43 (42.6%)
SYN	42 (41.6%)
CDX2	24 (30.7%)
CK	10 (12.8%)
GEP origin confirmation^d^	10 (90.9%)
Exclusion of NEN diagnosis	2 (2%)

^a^Among the 81 pts in whom Ki67 was available in the initial histological report

^b^Among the 85 pts in whom grading was available in the initial histological report

^c^Among the 75 pts in whom tumor differentiation was available in the initial histological report

^d^Among the 11 occult primaries at initial diagnosis

Overall, 78 (77.2%) patients needed additional immunohistochemical staining, particularly for CgA, Syn, CDX2, and CK, to confirm the neuroendocrine origin of the tumors and to detect the primary tumor site in those considered occult at initial diagnosis. According to the additional markers performed, an exclusion of NEN diagnosis was established in 2 (2%) patients: one patient had a diagnosis of colon adenocarcinoma with a microsatellite instability pattern, whereas the other patient had a diagnosis of breast cancer. Among the 11 patients with an initially unknown primary tumor site before referral to the center, the GEP origin was confirmed in 10 (90.9%) patients after additional immunohistochemical assessments, (Fig. 1). Among these confirmed GEP patients, 8 showed positivity at the CDX2 marker, confirming the suspicion of an intestinal origin, whereas the other 2 tumors, which were CDX-2 negative, were considered of pancreatic origin based on positivity at Isl-1. The primary tumor origin was subsequently confirmed by imaging procedures in all patients during the follow-up.

**Fig. 2:**
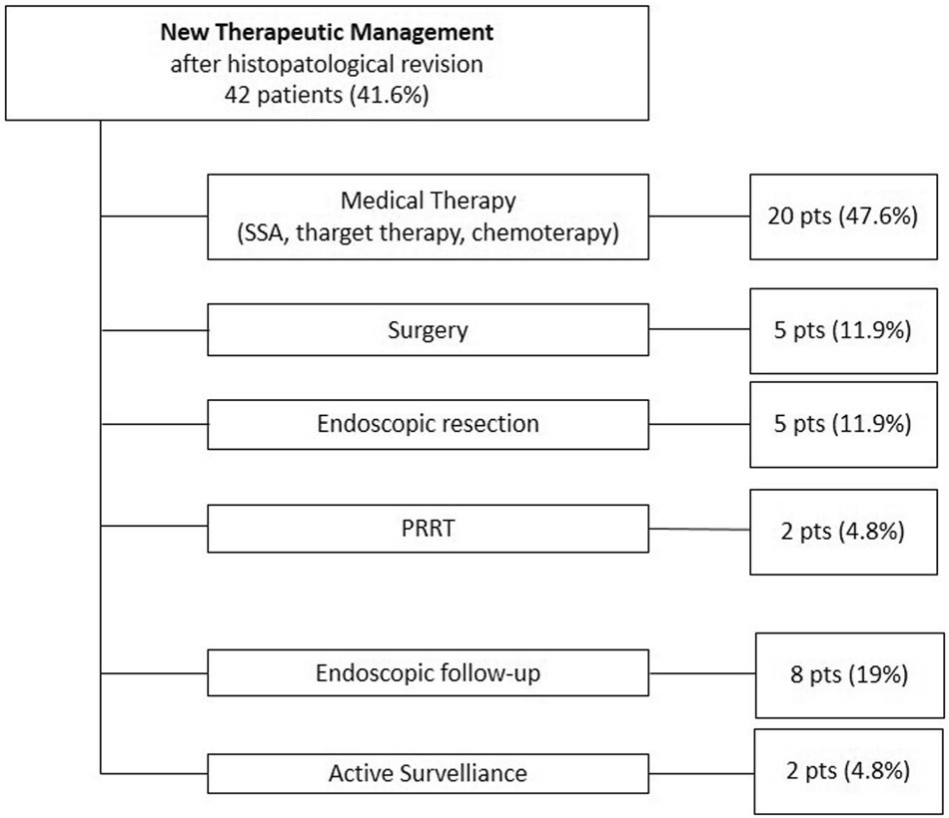
Impact of the histological revision on the clinical management

The clinical impact of the histopathological revision was considered when the NEN-dedicated MDT proposed a change in the patient’s therapeutic management after the pathological re-evaluation. Overall, this occurred in 42 (41.6%) patients, with 11 (26.2%) pNEN, 24 (57.1%) GI NEN (6 appendix, 2 colon, 2 duodenum, 5 rectum, 3 ileum, 6 stomach) and 7 (16.6%) neoplastic lesions of unknown origin at the initial diagnosis. The new therapeutic management proposed after the histological revision included i. medical therapy (SSA, target therapy or chemotherapy) in 20 (47.6%) patients (including the 2 patients in whom a diagnosis of NEN was excluded and who were started on chemotherapy); ii. surgery in 5 (11.9%) patients, including all stage II appendiceal NENs with changes in microscopic invasion evaluation after pathological reassessment; iii. endoscopic resection in 5 (11.9%) patients (including 3 confirmed stage I G1 rectal NEN, 1 small G1 duodenal NEN and 1 G2 g-NEN defined as type I after pathological reassessment); iv. peptide radionuclide therapy (PRRT) in 2 patients (4.8%) with an unknown primary tumor site at the initial diagnosis that was later confirmed to be of GEP origin after histopathological re-evaluation; v. endoscopic follow-up in 8 (19%) patients, including 5 type I g-NEN, 1 confirmed G1 duodenal NEN and 2 rectal NEN downstaged from G2 to G1 after pathological reassessment; vi. active surveillance in 2 (4.8%) patients, with 1 stage II appendiceal NEN without high-risk features at histopathological revision and 1 pancreatic NEN that was considered radically resected (Fig. 2).

In logistic regression, the presence of stage II disease was the only feature significanlty associated with changes in clinical management after histological revision (OR 5.83; *p* = 0.01).

The stage II patients in whom a histopathological revision had a significant impact on the clinical management included 5 patients with appendiceal NENs, in whom hemicolectomy was proposed after the evidence of high-risk features at the pathological reassessment, 2 patients with gastric NENs defined as type I after pathological reassessment and managed by endoscopic resection, 1 patient with a pancreatic NEN that was curatively resected and who underwent active surveillance, 1 patient with an ileal NEN treated with SSA after diagnosis and grading confirmation, and 1 patient with duodenal NEN managed by endoscopic follow-up after re-evaluation of resection margins and grading confirmation. Finally, a comparison of the patient features at the initial diagnosis according to the clinical impact after histological revision is described in Table 3.

**Table 3:** Comparison between patients with and without clinical impact after histological revision

Feature at the initial diagnosis	Clinical impact YES *n* = 42 (41.6%)	Clinical impact NO *n* = 59 (58.4%)	*P* value
Primary site			
Gastrointestinal	24 (36.9%)	41 (63.1%)	0.241
Pancreas	11 (44%)	14 (56%)	
Unknown	7 (63.7%)	4 (36.3%)	
Tumor stage			
I	11 (34.4)	21 (65.5%)	0.002
II	10 (76.9%)	3 (23.1%)	
III	2 (11.8%)	15 (88.2%)	
IV	19 (48.7%)	20 (51.3%)	
Pathological specimen			
Surgical or endoscopic resection	23 (47.9)	25 (52.1%)	0.232
Biopsy	19 (35.8%)	34 (64.2%)	
Site of pathological specimen			
Primary tumor	27 (39.1%)	42 (60.9%)	0.518
Metastasis	15 (46.9%)	17 (53.1%)	
Grading^a^			
G1	13 (38.2%)	21 (61.8%)	0.718
G2	19 (45.3%)	21 (35.6%)	
G3	5 (11.9%)	6 (10.2%)	
Differentiation^b^			
Well-differentiated	23 (38.3%)	37 (61.7%)	0.873
Moderately- differentiated	3 (30%)	7 (70%)	
Poorly- differentiated	2 (40%)	3 (60%)	

^a^Not available in 16 pts

^b^Not available in 26 pts

## Discussion

Although histopathological revision is known to provide a clinical benefit for the management of patients with different kinds of tumors, data on its utility in NENs are scant.

A need for standardized pathological reporting is highlighted by the ENETS, which encourages NEN-dedicated centers to standardize NEN pathological reporting [18]. Interestingly, the present study shows that essential items were missing in the pathological reports of the initial diagnosis in a significant proportion of the patients referred to our Center of Excellence. Specifically, in 20 patients (19.8%), data on Ki67, which is well known as the most important prognostic factor as well as the key factor able to drive clinicians for the optimal therapeutic choice [19, 20], were missing.

In addition to proliferative activity, it is recommended that pathologists report some mandatory immunohistochemical markers for a correct diagnosis of neuroendocrine tumors [5]*;* however, this study shows that at the initial diagnosis, an immunohistochemical evaluation was performed in only 46 (45.5%) patients. Furthermore, during the histological revision, an additional immunohistochemical evaluation (e.g., CDX2, and CK) was necessary in 78 (77.2%) patients.

An interesting finding was that the clinical impact after histopathological revision was more significant in stage II patients. At this intermediate stage, histopathological revision might provide useful information to properly plan clinical management, allowing clinicians to decide the most tailored therapeutic strategies. Interestingly, 5 appendicular NEN patients who were classified as stage II and were initially considered cured after appendectomy underwent right hemicolectomy after identifying high-risk features identified by the pathological reassessment. Appendicular NENs still represent a challenge for clinicians dealing with NENs, and a typical scenario is in which an accurate assessment of specific histological features is necessary to decide the best clinical approach [21]. In fact, the description of high-risk factors (such as the depth of extension into the mesoappendix, positive resection margins, perineural or lymphovascular invasion) is crucial for evaluating whether there is a surgical indication [22, 23]. As reported in a large Italian multicenter study, tumor size >15.5 mm, grading G2, and presence of lymphovascular infiltration were factors independently related to nodal metastases in appendiceal NENs [24]. However, a recent large European study showed that regional lymph node metastases of appendiceal NENs seem clinically irrelevant without a significant impact on patient survival [25]. These findings further corroborate the need for an accurate histopathological analysis in order to identify risk factors affecting clinical outcome, which, as suggested by the results of the present study, should be performed in a dedicated NEN center.

A particular utility of the histological revision was observed in those tumors initially classified as of unknown primary origin. In fact, as a consequence of the histological revision, a specific immunohistochemical pattern suggesting the origin of the primary tumor was observed in the majority of cases (10 out of 11). This finding plays a significant clinical role, given the worse prognosis that affects patients with unknown primary tumors (12–22% of NEN diagnoses) owing to the more advanced stage at the time of diagnosis and the potential delay in starting a specific medical treatment for these patients [9, 26].

Other studies have reported the utility of implementing the immunohistochemical profile in unknown primary NENs. In a recent study, a marker panel including TTF1, CDX2, and Isl-1 predicted the primary site in 6 out of 10 NENs of unknown origin, reducing their rate from 12% to 5% [26]. In a large NEN series of well-differentiated GI NENs, CDX2 showed high sensitivity (89%) and specificity (94%) for NENs of the jejunoileum and appendix. Moreover, immunohistochemical detection of CDX2 has been demonstrated in 90% of primary and 91% of metastatic jejunoileum NENs [27].

Another important finding of this study was that after histopathological revision and discussion in a NEN-dedicated MDT, a change in clinical management occurred in 42 (41.6%) patients. The potential utility of the histological review in a dedicated NEN center was previously reported by a multicenter retrospective study [28]. In that study, it was reported that histological revision had a clinical impact in 36% of patients. However, a direct comparison between that paper and the present study is not properly feasible, given the multicenter design of that study, which could affect the results in terms of heterogeneity in the immunohistochemical assessment (particularly for Ki-67), and because of the nonstandardized clinical approaches used by different multidisciplinary teams in different centers. Multidisciplinary care is strongly encouraged by both the European and North American Neuroendocrine Tumor Society [22, 29]. In a previous experience, we observed that after MDT discussion, the clinical management changed in 50.3% of the patients, and integration of pathological data, including histological revision or new bioptic sampling, was needed in 43.1% of them [15].

Although we believe that data from the present study may contribute to increasing the knowledge in the management of NEN patients, we are also aware of the study limitations, including the relatively low number of patients included, the retrospective study design (which is an intrinsic weaknesses of most studies evaluating NENs because of the rarity of this disease), and the lack of an external histological review able to confirm the results obtained by our histopathological assessment, which was performed by an experienced NEN specialist pathologist working in the center of excellence for many years.

## Conclusion

Histopathological revision in a referral NEN center by an expert pathologist provides relevant information useful for clinicians dealing with NEN to obtain the correct diagnosis and to plan the optimal therapeutic approach, and it should be performed in those patients in whom the initial diagnosis was given in a nonexperienced center. Early referral to a NEN-dedicated center may shorten the delay in diagnosis and increase the opportunity for patients to receive the best care.

## Data Availability

The data that support the findings of this study are available on request from the corresponding author
